# Statistical Approaches Used to Assess the Equity of Access to Food Outlets: A Systematic Review

**DOI:** 10.3934/publichealth.2015.3.358

**Published:** 2015-07-28

**Authors:** Karen E. Lamb, Lukar E. Thornton, Ester Cerin, Kylie Ball

**Affiliations:** 1 Centre for Physical Activity and Nutrition Research, School of Exercise and Nutrition Sciences, Faculty of Health, Deakin University, 221 Burwood Highway, Burwood, VIC, 3125, Australia.

**Keywords:** food environment, neighbourhood, socio-economic status, statistical methods, spatial autocorrelation, spatial statistics

## Abstract

**Background:**

Inequalities in eating behaviours are often linked to the types of food retailers accessible in neighbourhood environments. Numerous studies have aimed to identify if access to healthy and unhealthy food retailers is socioeconomically patterned across neighbourhoods, and thus a potential risk factor for dietary inequalities. Existing reviews have examined differences between methodologies, particularly focussing on neighbourhood and food outlet access measure definitions. However, no review has informatively discussed the suitability of the statistical methodologies employed; a key issue determining the validity of study findings. Our aim was to examine the suitability of statistical approaches adopted in these analyses.

**Methods:**

Searches were conducted for articles published from 2000–2014. Eligible studies included objective measures of the neighbourhood food environment and neighbourhood-level socio-economic status, with a statistical analysis of the association between food outlet access and socio-economic status.

**Results:**

Fifty-four papers were included. Outlet accessibility was typically defined as the distance to the nearest outlet from the neighbourhood centroid, or as the number of food outlets within a neighbourhood (or buffer). To assess if these measures were linked to neighbourhood disadvantage, common statistical methods included ANOVA, correlation, and Poisson or negative binomial regression. Although all studies involved spatial data, few considered spatial analysis techniques or spatial autocorrelation.

**Conclusions:**

With advances in GIS software, sophisticated measures of neighbourhood outlet accessibility can be considered. However, approaches to statistical analysis often appear less sophisticated. Care should be taken to consider assumptions underlying the analysis and the possibility of spatially correlated residuals which could affect the results.

## 1. Introduction

Obesity is one of the leading public health concerns globally and is linked to a number of health conditions, such as cardiovascular disease and type 2 diabetes. Individual-level interventions have had limited success in curbing rising rates of overweight and obesity [1],[2]. In recent years, research has examined environmental factors which may influence weight gain, in particular, the “obesogenic environment”, defined as an environment which facilitates unhealthy behaviours, such as poor diet, and provides limited opportunities to engage in healthy activities, such as physical activity [3]. The environment has been posited as a contributing factor to the higher levels of obesity observed amongst those residing in more socioeconomically disadvantaged areas [4]. In particular, disadvantaged areas have been investigated for the presence of higher levels of fast food outlets [5],[6] and reduced access to healthier outlets, such as supermarkets or grocery stores [7]–[9].

In a recent review of ten semi-systematic and systematic review articles, nine of which considered disparities in access to food outlets by neighbourhood-level socio-economic status (SES), Black et al. [10] found that although these reviews tended to suggest food deserts exist in the US, with those living in low SES neighbourhoods identified as having lower access to supermarkets [11]–[14] and often greater access to fast food outlets [12],[13],[15]–[17], findings from other countries have been equivocal [10]. Such equivocal findings, while potentially due to true differences in these diverse built environment contexts, may also in part be explained by a number of methodological factors, including inappropriate analytical methods or inconsistent approaches when accounting for the spatial autocorrelation in the studies.

In the nine reviews of disparities in access to food outlets, few explicitly discussed the statistical methods employed to examine the associations. When mentioned, these reviews almost exclusively only discussed limitations attributable to the cross-sectional design common to published studies, highlighting that these approaches mean that causal inference cannot be made [12],[13],[15],[17],[18]. In their review of access to fast food outlets, Fraser et al. [16] mentioned that the statistical methods in the studies examined were typically simple approaches, such as correlation or simple regression, but did not discuss how appropriate these methods were for the questions being addressed or the structure of the data being considered. The choice of statistical methodology adopted is important as using an inappropriate methodology can lead to incorrect findings [19]. It is important for researchers to verify the assumptions underlying the methodology undertaken to ensure that the method is suitable. For example, a two-sample t-test can provide misleading results if it is adopted when the data are not normally distributed and the groups have unequal variance [20]. Thus, in this situation an alternative statistical method would be more suitable to test for differences between groups.

In another review, Fleischhacker et al. [15] discussed analytical considerations in studies of the distribution of food outlets, such as a lack of detail in the methodology as to how population adjustment was conducted. Importantly, the authors identified four key factors which should be developed further in studies of the fast food environment and its effect on health and behaviour outcomes: software, statistics, sample size, and the size/range of the neighbourhood buffers. While studies have compared the size and range of buffers of food access measures [21], important statistical considerations when dealing with spatial data in this field have not been addressed. In one review of the distribution of fast food outlets, Fraser et al. [16] stated that alternative statistical approaches such as geographically weighted regression, a technique for exploring how relationships vary in space [22], could be utilised in these studies but did not describe the benefits of adopting this technique or mention any other spatial statistical techniques or considerations.

In one review examining associations between the community food environment, defined as the ‘number, type, location and accessibility of food outlets’ in an area [23], and obesity, Holsten [24] highlighted that “since objects are spatially related and not independent, many analyses should have controlled for spatial autocorrelation”. Spatial autocorrelation refers to the degree of similarity of neighbouring observations. Although this is an important methodological issue when examining the equity of access to outlets across neighbouring areas where correlation is likely to be present, it was not discussed in any reviews of the distribution of food outlets. Ignoring spatial autocorrelation through the use of typical parametric statistical techniques, such as linear regression, can lead to erroneously identifying statistically significant associations when in fact none exist or, alternatively, to failing to identify associations when they are present [25]–[27]. In a review of ecological studies which compared analyses with and without adjustment for spatial autocorrelation, Dormann [28] discussed some of the consequences of ignoring spatial autocorrelation on model parameters, namely obtaining biased parameter estimates and “overly optimistic” standard errors, and found that in all studies reviewed the coefficients were affected by spatial autocorrelation. Therefore, the choice of statistical analysis technique employed and the degree of spatial autocorrelation can influence research findings.

The aim of this review was to systematically appraise the existing literature on the equity of access to food outlets to identify the statistical methods used in the analyses. The key focus was to examine the suitability of the methodology employed and to identify any spatial statistical methodologies used. The secondary aim was to assess whether or not spatial autocorrelation was considered.

## 2. Materials and Method

### 2.1. Inclusion criteria

Articles were eligible for inclusion if they featured an objective measure of the neighbourhood food environment considered as an outcome variable in the analysis. Included studies contained a measure of neighbourhood-level SES (e.g., median household income, socioeconomic index for areas). Papers were excluded if they solely examined within-store produce as an outcome, rather than store availability, such as those which examined healthy food baskets or shelf space use, or if the focus was on dietary or obesity outcomes rather than store availability or distribution. Furthermore, articles were excluded if they did not conduct a formal statistical analysis of the association between neighbourhood-level SES and food store access; that is, studies which only produced descriptive tables or maps of the distribution but did not attempt to identify evidence of an association between food store access and neighbourhood-level SES through statistical tests.

### 2.2. Search strategy

The electronic search was conducted in March 2014 and the search strategy adopted is fully specified in the Appendix. Our search included journal articles published in English since 2000 as existing reviews of food environment literature have shown that the majority of environmental food assessments have occurred during this period [15],[17]. Articles were identified using the following databases: Medline Complete, PsychINFO, CINAHL Complete, Web of Science, Global Health, Embase, Scopus and the Cochrane Library. Relevant articles known to the authors were examined to identify key words to use as search terms. Our search terms included combinations of terms referring to food outlets, equity and neighbourhood access as detailed in the Appendix.

Initially, a title scan was conducted in order to discard irrelevant articles identified in the search. A two stage process was adopted when screening abstracts. First, review articles, commentary or discussion articles and intervention studies (in which the focus was on individual outcomes), studies which examined students' diets or the school food environment, and any other studies which did not involve an objective measure of the food environment were excluded. In the second stage, two investigators (KEL and LET) independently assessed the remaining abstracts according to the inclusion criteria to compile a final list of articles. Where there was disagreement, the full article was examined and discussed, with input from all co-authors, to identify if this should be included in the review.

### 2.3. Data extraction

A structured form was created for the data extraction which included information on where the study was conducted, the number of neighbourhoods considered, the statistical analysis approach adopted (including whether or not spatial autocorrelation was considered) and the main findings.

## 3. Results

**Figure 1.**
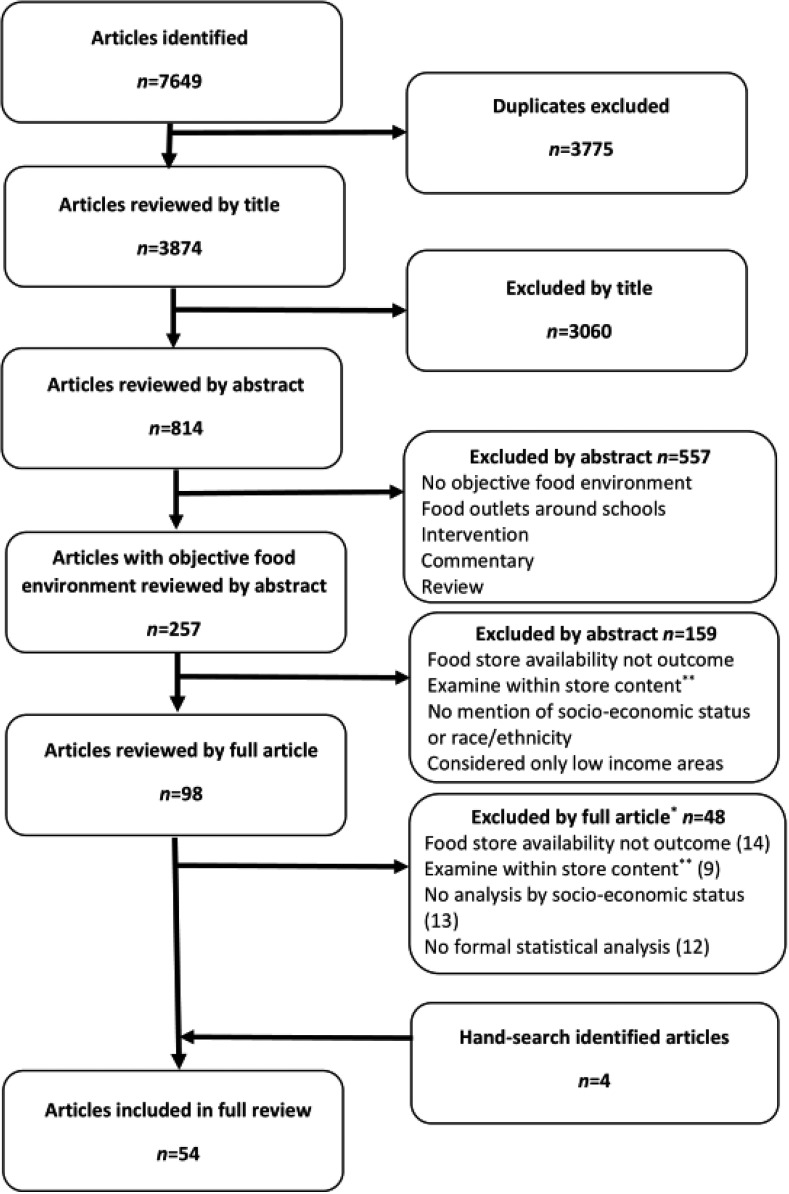
Flow chart summary of articles identified in literature search and included in the review. * Articles can appear in more than one category. Numbers excluded by full article represent primary exclusion reason. ** This includes shelf-space/display, produce availability, price, quality and marketing

The results from the search are presented in Figure 1. A total of 54 published papers were considered in this systematic review.

## 3.1. Summary of included studies

The 54 included papers, described in Table 1, published between 2002 (no articles published in 2000 and 2001 met the inclusion criteria) and 2014 feature studies of food access and availability from the US (n = 26; 48.1%), Canada (n = 10; 18.5%), the UK (n = 7; 13.0%), New Zealand (n = 4; 7.4%), Australia (n = 3; 5.6%), Brazil (n = 1; 1.9%), Denmark (n = 1; 1.9%), Germany (n = 1; 1.9%), and Sweden (n = 1; 1.9%). The median sample size (i.e., number of administrative units) was 390, although there was a great deal of variability (IQR = 5671.8) and two articles did not report sample sizes [29],[30]. The samples ranged from as low as 18 neighbourhoods in one article which examined fast food outlet availability in Cologne, Germany [31] to as high as 65,174 in a recent article which considered the availability of supermarkets, grocery and convenience stores across census tracts for the whole of the US [32]. Just under one third of the articles (n = 16; 29.6%) involved studies of more than 1000 neighbourhoods and the majority of these (n = 12) were national, or urban national studies, while the others (n = 4) were US city or county studies.

Included articles considered a wide variety of food outlet types, such as fast food outlets, supermarkets or grocery stores (typically defined as smaller supermarkets and / or non-chain supermarkets), convenience stores, green grocers, cafés, specialty food stores (e.g., meat markets, fishmongers), and delicatessens. Of these, the most commonly considered outlet types were supermarkets and fast food outlets, with some analyses considering the distribution of both outlet types.

Although the primary purpose of this review was to examine the statistical techniques employed, we have highlighted the key study findings in Table 1. As in other systematic reviews [10],[15],[16], findings relating to the distribution of supermarkets and grocery stores by neighbourhood-level SES were mixed while results relating to fast food outlet distribution were more consistent, particularly in the US, with greater availability in low SES areas. Findings from the studies which examined the distribution of other food store types varied (Table 1).

**Table 1.** Summary of included articles (n = 54).

Lead author, Year [ref. no.]	City/Region, Country	Neighbour-hood definition, number	Food store	SES measure*	Key findings relating to neighbourhood SES^†^
Anchondo, 2011 [35]	El Paso County, Texas, USA	Census tracts, N = 126	i) Supermarkets (chain); ii) Grocery stores; iii) Specialty stores (bakery, fruit, vegetable, meat markets); iv) Convenience stores.	PCA* used to combine: % households below poverty level; % adults >25 years with low education;median tract income; % households with public assistance income; % households with >1 person/room; % of individuals employed in professional/managerial occupations; % households with no vehicle access; % adults unemployed and actively seeking work; % female head of household with children. Index standardised to have mean 0 and variance of 1. Split into high (top 25%), intermediate (50%), and low (bottom 25%).	i) Supermarkets more common in advantaged neighbourhoods; ii) Grocery stores more common in deprived neighbourhoods; iii) Specialty stores in more common more deprived neighbourhoods but no evidence of a difference in multivariate analysis; iv) Convenience stores more common in more advantaged neighbourhoods.
Apparicio, 2007 [48]	Montreal, Canada	Census tracts, N = 506	i) Supermarkets (major chain)	Low income population; Social deprivation index (sum of variables standardised to 0 to 1 scale: % low income people; % lone-parent families;unemployment rate; % aged > 20 years with low education; % recent immigrants.	i) Supermarket access increases with increasing deprivation.
Bader, 2010 [63]	New York City, USA	Census tracts, N = 2172	i) Supermarkets	Proportion of residents living below the federal poverty line split into quartiles.	i) Density of supermarkets highest in most advantaged neighbourhoods.
Baker, 2006 [45]	Urban St Louis County, USA	Census tracts, N = 270	i) Supermarkets and grocery stores (chain); ii) fast food outlets (chain).	% living below US federal poverty level grouped into three categories: <10%, 10-19.9%, 20%+.	i) & ii) High deprivation neighbourhoods are less likely to have access to food outlets than more advantaged neighbourhoods.
Ball, 2009 [52]	Melbourne, Australia	Suburbs, N = 45	i) Fruit and vegetable grocery stores; ii) Supermarkets (major chain).	Socio-Economic Index For Areas (SEIFA) split into low, mid, high levels of SES.	i) Higher density of fruit and vegetable stores in more deprived neighbourhoods; ii) Higher density of supermarkets in more deprived neighbourhoods.
Berg, 2008 [64]	Dallas County, Texas, USA	Block groups, N = 1681	i) Grocery stores (chain)	Median neighbourhood income. Number of clients on HHSC programs.	i) More common to have no stores in neighbourhoods of lower income and with higher numbers of clients on HHSC programs.
Black, 2011 [40]	British Columbia, Canada	Census tracts, N = 630	i) Supermarkets; ii) Supermarkets, grocers, food markets, fruit and vegetable stores, independent seafood, meat, poultry, milk and cheese stores.	Median household income.	i) Fewer supermarkets with increasing income; ii) Fewer supermarkets and fresh food stores with increasing income.
Block, 2004 [5]	New Orleans, USA	Census tracts, N = 156	i) Fast food restaurants (chain).	Median household income.	i) Number of fast food outlets decreased with increasing income. Association not significant after adjustment for race.
Bower, 2014 [32]	USA	Census tracts, N = 65,174	i) Supermarkets; ii) Grocery stores; iii) Convenience stores	% living below US federal poverty level grouped into three categories: <10%, 10-19.9%, 20%+.	i) Number of supermarkets decreases with increasing deprivation; ii) Number of grocery stores increases with increasing deprivation; iii) Number of convenience stores increases with increasing deprivation.
Burns, 2007 [57]	Casey, Melbourne, Australia	Census districts, N = 244	i) Supermarkets (major chain); ii) Fast food outlets (major chain).	Socio-Economic Index For Areas (SEIFA).	i) Supermarkets closer with increasing affluence; ii) Fast food outlets closer with increasing deprivation.
Cubbin, 2012 [58]	Alameda County, California, USA	Census tracts, N = 321	i) Healthy outlets (fruit and vegetable markets, grocery stores, food markets); ii) Unhealthy outlets (fast food, pizza places, convenience stores).	% with income below US federal poverty level split into three poverty trajectories: stable, affluent; stable, moderate poverty; stable, concentrated poverty.	i) Long-term poverty neighbourhoods have greatest access to healthy outlets; ii) Long-term poverty neighbourhoods have greatest access to unhealthy outlets.
Cummins, 2005 [6]	England & Scotland, UK	Super output areas, N = 32,482 & Data zones, N = 6505	i) Fast food outlets (McDonald's)	Index of Multiple Deprivation. Continuous measure of compound social and material deprivation calculated using a variety of data including current income, employment, health, education and housing. Grouped into quintiles.	i) Greater mean numbers of McDonald's with increasing deprivation.
Cushon, 2013 [29]	Saskatoon, Saskatchewan, Canada	Residential blocks, N = not reported	i) Supermarkets (major chains); ii) Fast food outlets.	Deprivation index. Two dimensions: social and material. Social deprivation consists of proportion of lone parents, proportion of residents living alone and marital status. Material deprivation consists of educational attainment, average income and employment status. Grouped into quintiles.	i) Distance to the nearest supermarket further for most deprived quintile according to material disadvantage but further for least deprived quintile of social deprivation; ii) Most deprived quintile closer to fast food outlets when considering material deprivation but proximity to the nearest increased with increasing social deprivation.
Dai, 2011 [59]	Mississippi, USA	Census tracts, N = 121	i) Food stores (supermarket, grocery, convenience, meat and fish, fruit and vegetable, candy and nut, dairy, bakery, natural food and specialty; excluding restaurants, school or work place cafeterias, and other food providers).	PCA* used to combine: female-headed household; occupied house ownership; median household income; carless occupied household; linguistically isolated household; non-white population; household lacking complete plumbing facilities; population (aged ≥25yrs) without high school diploma; population (aged ≥17yrs) below poverty level; household lacking kitchen facilities; occupied house with >1 occupant per room; rural population. Combined in three independent factors (rural population spread loadings across all three): urban socioeconomic disadvantage, rural socioeconomic disadvantage, and cultural barriers.	i) Greater access to food stores in more disadvantaged areas.
Daniel, 2009 [65]	Montreal, Canada	Census tracts, N = 846	i) Healthy food stores (fruit and vegetable stores, supermarkets and grocery retail stores, farm markets); ii) Fast food outlets (chain).	Median household income.	i) No association between median household income and healthy food stores; ii) No association between median household income and fast food outlets.
Gordon, 2011 [66]	New York, USA	Block groups, N = 448	i) Supermarkets; ii) Healthy bodegas; iii) Fast food restaurants; iv) Food desert index.	Median household income	i) Higher proportion of supermarkets in higher income areas; ii) Higher proportion of healthy bodegas in higher income areas; iii) Lower proportion of fast food outlets in higher income areas; iv) Higher food desert index in higher income areas.
Gould, 2012 [67]	Gatineau, Quebec, Canada	Dissemination areas, N = 392	i) Supermarkets; ii) Food stores; iii) Area devoted to fresh fruit and vegetable sale (≥7m^2^ of shelf and floor space).	Proportion separated/divorced/widowed; proportion of single-parent families;proportion of individuals aged 24-65yrs without high school diploma; employment rate (%); median household income before tax ($). Proportions for each variable were scaled between 0 and 1 and employment rate and income were inverted to insure they vary in accordance with deprivation. The index was divided into quartiles.	i) Distance to nearest supermarket decreases with increasing deprivation; ii) Distance to nearest food store decreases with increasing deprivation; iii) Greater fresh fruit and vegetable availability with increasing deprivation.
Hemphill, 2008 [68]	Edmonton, Alberta, Canada	Municipally defined units, N = 204	i) Fast food outlets.	Proportion of low-income individuals; proportion of individuals without a high school diploma; proportion unemployed; proportion renting; proportion recent immigrants.	i) Fast food outlet availability increased with increasing proportions of low-income individuals, increasing proportions of unemployed individuals; increasing proportion of renters. Differences were identified in the number of fast food outlets by proportion of individuals without a high school diploma and the proportion of recent immigrants but the results did not follow a pattern of increased access with increasing proportion.
Hill, 2012 [50]	City of Danville, Dan River region, USA	Block groups, N = 39	i) Food stores (grocery, convenience); ii) Restaurants (fast casual restaurant, fast food outlet, sit down restaurant).	Median family income split into deciles and grouped as low income (deciles: 1-4), middle (5-6), high (7-10).	i) No evidence of a difference in food stores by median income; ii) Greater average number of restaurants available in middle income areas.
Howard, 2007 [51]	Santa Cruz, Monterey, and San Benito Counties, California, USA	Census blocks, N = 6308	i) Food retail outlets selling fruit and vegetables	Median household income.	i) Outlet density increased with decreasing median household income.
Hurvitz, 2009 [38]	King County, USA	Census tracts, N = 373	i) Fast food outlets (chain and non-chain)	Median household income.	i) Greater number of fast food outlets in low income neighbourhoods.
Jaime, 2011 [46]	Sao Paulo, Brazil	Sub-municipalities N = 31	i) Supermarkets (chain); ii) Grocery stores; iii) Fruit and vegetable specialised food markets. iv) Total retail food store density; v) Fast food restaurants.	Human Development Index of the area. Combines normalised measures: life expectancy; educational attainment; average per capita income of the area. Varies from 0 to 1. Grouped into tertiles.	i) Supermarkets more prevalent in least deprived areas; ii) Grocery stores more prevalent in least deprived areas; iii) Specialised food markets more prevalent in least deprived areas; iv) Food stores more prevalent in least deprived areas; v) Fast food restaurants more prevalent in least deprived areas.
Jones, 2009 [69]	Nova Scotia, Canada	Communities, N = 266	i) Fast food outlets (chain)	PCA* used to combine z-scores of age-sex standardised: average individual income (≥15yrs old); unemployment rate (≥25yrs old); <high school diploma (≥25yrs old); Material deprivation defined by adding standardised variable scores for these variables, multiplied by their respective weights. Scores were split into quintiles.	i) Mean number of fast food outlets increases with decreasing deprivation.
Kawakami, 2011 [39]	Urban Sweden	Small area market statistics, N = 6986	Relevant to food sales: i) Food/grocery stores (all/chain/non-chain); ii) Convenience stores; iii) Gas station food/grocery stores; iv) Restaurants; v) Fast food restaurants.	Created and summed z-scores of each of: low income; unemployment; low education; social welfare recipient status. All for those aged 25-64yrs. Index split into low (<1 SD from mean), medium (within 1 SD from mean), high (>1 SD from mean).	i)-v) Moderate and high deprivation areas had higher availability of food/grocery stores, convenience stores, gas station food/grocery stores, restaurants, and fast food restaurants.
Kwate, 2009 [44]	New York City, USA	Census block groups, N = 5730	i) Fast food outlets (chain)	Median household income.	i) No strong effect of household income on fast food outlet availability. High income Black areas had similar exposure to low income Black areas.
Larsen, 2008 [70]	London, Ontario, Canada	Census tracts, N = 76	i) Supermarkets	Considered separately and summed z-scores: proportion that have not graduated from high school; proportion of lone parent families versus the total number of families; unemployment rate; proportion of households that fall below the low income cut-off according to Statistics Canada. Summed score was split into low distress, moderate distress, and high distress for analysis.	i) Most distressed areas had lowest access to supermarkets by walking and least distressed areas had highest; middling areas of distress had lowest access when considering access by public transit; no evidence of a difference by neighbourhood distress when considering number accessible within 1000m; no evidence of a difference when considering distance to the nearest supermarket.
Lee, 2009 [30]	Buffalo, New York, USA	Census block groups, N = not reported	i) Grocery stores	Number of families whose income falls below the poverty level.	i) Mid-eastern part of the city suffers from a lack of grocery store provision.
Lisabeth, 2010 [43]	Nueces County, Texas, USA	Census tracts, N = 64	i) Supermarkets (chain); ii) Grocery stores; iii) Convenience stores; iv) Meat, seafood and produce specialty stores.	Median income.	i) No association between median income and supermarkets; ii) Number of grocery stores decreases with increasing income; iii) Number of convenience stores decreases with increasing income; iv) Number of specialty stores decreases with increasing income.
Macdonald, 2007 [71]	England and Scotland, UK	Super output areas, N = 32,482 & Data zones, N = 6505	i) Fast food outlets (chain: McDonald's, Burger King, KFC, Pizza Hut)	Index of multiple deprivation. A continuous measure which includes income, employment, health, education and housing. Split into quintiles.	i) Number of fast food outlets greater in more deprived areas. However, the association did not follow a straightforward trajectory whereby outlets increased with increasing deprivation.
Macdonald, 2009 [72]	Glasgow, UK	Data zones, N = 694	i) All food retailers; ii) Bakers; iii) Butchers; iv) Fruit and vegetable stores; v) Fishmongers; vi) Convenience stores; vii) Supermarkets; viii) Delicatessens.	Income sub-domain of Scottish index of multiple deprivation. Based on numbers of residents claiming a range of financial welfare benefits. Split into Glasgow-based quintiles.	i) Number of all food outlets roughly increases with increasing deprivation. Distance to the nearest outlet greatest in least deprived areas but no clear trend in association. ii) No evidence of a difference in mean number of bakers by deprivation. Some evidence of a difference in mean distance but no clear trend. Mean distance to nearest baker furthest in most deprived neighbourhoods. iii) No clear trend in association between deprivation and butcher access- second least deprived neighbourhoods had highest average number of butchers. No evidence of a difference in mean distance to nearest butcher. iv) No evidence of a difference in mean number of fruit and vegetable stores or mean distance to the nearest store by deprivation. v) No evidence of a difference in mean number of fishmongers by deprivation. Average distance to the nearest increases with increasing deprivation. vi) No clear trend in association between deprivation and convenience store access but most deprived neighbourhoods had highest mean number of stores. No clear trend in association between average distance to nearest convenience store and deprivation but distance greatest for least deprived neighbourhoods. vii) No evidence of a difference in mean number of supermarkets by deprivation. Difference in mean distance to nearest supermarket by deprivation, with distance roughly increasing with increasing deprivation. viii) No evidence of a difference in mean number of delicatessens by deprivation. Association between deprivation and distance to nearest delicatessen but no clear trend- average distance highest in second most deprived neighbourhoods.
Macintyre, 2008 [73]	Glasgow, UK	Data zones, N = 694	Relevant to food sales: i) Supermarkets; ii) Fast food outlets (chain); iii) Cafés.	Income sub-domain Scottish index of multiple deprivation; based on numbers of residents claiming a range of financial welfare benefits. Split into Glasgow-based quintiles.	i) No evidence of a difference in number of supermarkets by deprivation. Weak (*p* = 0.06) evidence of a difference in distance to nearest supermarket by deprivation: average distance increases with increasing deprivation. ii) No evidence of a difference in the number of fast food outlets by deprivation. No evidence of a difference in distance to the nearest outlet by deprivation. iii) No evidence of a difference in the number of cafés by deprivation. Average distance to the nearest café increases with increasing deprivation from Q2 to Q5.
Macintyre, 2005 [47]	Glasgow, UK	Data zones, N = 694	i) Restaurants (independent and chain restaurants); ii) Fast food outlets (chain); iii) Cafés; iv) Takeaway.	Data zone level Scottish index of multiple deprivation; based on current income, employment, health, education, skills and training, telecommunications, and housing. Split into quintiles.	i) Evidence of an association between number of restaurants and deprivation but no clear trend. Highest access in second most affluent area. Second most affluent area has greater odds of having a restaurant than middling and deprived areas. ii) No evidence of a difference in fast food outlet number by deprivation, or in odds of having a fast food outlet. iii) No evidence of a difference in number of cafés by deprivation. Odds of the presence of a café are lower in the second most deprived quintile than the second most affluent. iv) Evidence of an association between deprivation and the number of takeaways but no clear trend. Highest access in second most affluent area. Lower odds of having a takeaway outlet present in the most affluent quintile than the second most affluent.
Meltzer, 2012 [74]	New York City, USA	ZIP-codes, N = 208	Relevant to food sales: i) Supermarkets; ii) Pharmacies and personal care stores; iii) Food service establishments. iv) McDonald's; v) Subway; vi) Starbucks; vii) Dunkin Donuts.	Average household income (<80% vs. ≥80% of NYC average).	i) More grocery stores in low income areas; ii) Fewer drug stores in low income areas; iii) Fewer food service establishments in low income areas; iv) More McDonald's outlets in low income areas; v) More Subway outlets in low income areas; vi) More Starbucks outlets in middling-high income areas; vii) Dunkin Donuts more numerous in middling-high income areas.
Mercille, 2012 [75]	Montreal, Canada	Census tracts, N = 248	i) Fast food outlets (chain and non-chain); ii) Fruit & vegetable stores (groceries, supermarkets, fruit and vegetable stores, farmer's markets).	Proportion of households below the low-income threshold. Split into quartiles.	i) Fewer fast food outlets in lowest poverty areas but highest in second highest poverty area; ii) Higher number of fruit and vegetable outlets available in more deprived areas.
Molaodi, 2012 [42]	England, UK	Lower super output areas, N = 32,482	Relevant to food sales: i) Fast food outlets (chain); ii) Supermarkets (chain).	Income sub-domain of index of multiple deprivation. Split into quintiles.	i) Number of fast food outlets increased with increasing deprivation; ii) Number of supermarkets increased with increasing deprivation from Q1 to Q4 but was lower in the most deprived quintile than in Q4.
Moore, 2006 [34]	North Carolina, Maryland, & New York, USA	Census tracts, N = 685	i) Grocery stores and supermarkets; ii) Convenience stores; iii) Meat and fish markets; iv) Fruit and vegetable markets; v) Bakeries; vi) Natural food stores; vii) Specialty stores.	Median household income. Split into tertiles.	i) Number of grocery stores increases with increasing deprivation. Fewer supermarkets in low income areas than high income areas. ii) Number of convenience stores increases with increasing poverty. iii) Number of meat and fish markets increases with increasing poverty. iv) No clear differences in fruit and vegetable markets by income. v) Lower number of bakeries in the lowest income areas than highest. vi) Fewer natural food stores with increasing poverty. vii) Fewer specialty food stores with increasing poverty.
Morland, 2002 [36]	Jackson City, Mississippi; Forsyth County, North Carolina; Washington County, Maryland; selected suburbs of Minneapolis, USA	Census tracts, N = 216	i) Supermarkets (chain); ii) Grocery stores; iii) Convenience stores; iv) Convenience stores with gas stations; v) Specialty food stores (meat markets, fruit and vegetable markets); vi) Full-service restaurants (including cafeterias); vii) Fast food outlets (chain and non-chain); viii) Fast food outlets (chain); ix) Carryout eating places (non-chain delicatessens, bagel or sandwich shops); x) Carryout specialty items (smoothie shops, espresso bars, specialise in one type of food); xi) Bars/taverns.	Median value for homes. Site-specific quintiles of wealth were averaged to create a measure of relative wealth.	i) Supermarkets more prevalent in less deprived areas but no clear trend; ii) Grocery stores more prevalent in more deprived areas; iii) No clear evidence of a difference in convenience stores by derivation; iv) More convenience stores with gas stations in middling deprivation areas compared to high deprivation areas; v) No clear evidence of a difference in specialty food stores by deprivation; vi) No clear evidence of a difference in full-service restaurants by deprivation; vii) & viii) No clear evidence of a difference in fast-food outlets by deprivation; ix) No clear evidence of a difference in carryout outlets by deprivation; x) No clear evidence of a difference in specialty carryout outlets by deprivation; xi) Lower numbers of bars/taverns in the two most affluent quintiles than the least affluent neighbourhoods.
Pearce, 2007 [53]	New Zealand	Meshblocks, N = 38,350	i) Fast food outlets (chain and non-chain); ii) Supermarkets and locally operated convenience stores and service stations selling fresh food.	New Zealand deprivation index based on: car access; tenure; benefit receipt; unemployment; low income; telephone access; single-parent families; education; living space. Index split into deciles.	i) Median distance to nearest fast food outlet decreases from second most affluent to second most deprived decile; ii) Median distance to nearest supermarket decreases from second most affluent to second most deprived decile.
Pearce, 2008 [76]	Urban New Zealand	Meshblocks, N = 22,780	Relevant to food sales: i) Supermarkets; ii) Convenience stores (including service stations selling fresh food); iii) Fast food outlets (chain and non-chain).	New Zealand deprivation index based on nine socio-economic characteristics (e.g., car access, tenure and benefit receipt). Index split into quintiles.	i) Number of supermarkets increases with increasing deprivation; ii) Number of convenience stores increases with increasing deprivation; iii) Number of fast food outlets increases with increasing deprivation.
Pearce, 2007 [54]	New Zealand	Meshblocks, N = 38,350	Relevant to food sales: i) Food shops; ii) Supermarkets.	New Zealand deprivation index based on nine socio-economic characteristics.	i) & ii) Travel time decreased with increasing deprivation for both food shops and supermarkets.
Pearce, 2008 [55]	New Zealand	Meshblocks, N = 38,350	i) Supermarkets; ii) Food outlets	New Zealand deprivation index based on nine socio-economic characteristics.	i) & ii) Median travel times were greater in least deprived areas compared to most deprived for both supermarkets and food outlets.
Powell, 2007 [37]	USA	ZIP-codes, N = 28,050	i) Full service restaurants; ii) Fast food outlets	Median household income. Income quintiles: (<$29,066; ≥$29,066-<$34,291; ≥$34,291-<$40,049; ≥$40,049-<$49,905; ≥$49,905). Dichotomous indicators created for each category.	i) & ii) Higher income areas had lower numbers of full service restaurants and fast food outlets than lower income areas.
Powell, 2007 [9]	USA	ZIP-codes, N = 28,050	i) Supermarkets (chain); ii) Supermarkets (non-chain); iii) Grocery stores; iv) Convenience stores.	Median household income. Split into low (bottom quintile), middle (middle three quintiles), and high (top quintile).	i) Low income and high income areas have fewer chain supermarkets than middle income areas; ii) Low income areas have more non-chain supermarkets than middle income areas; iii) Low income areas have more grocery stores and high income areas have fewer grocery stores than middle income areas; iv) Low income areas have more convenience stores and high income areas have fewer convenience stores than middle income areas.
Reidpath, 2002 [77]	Melbourne, Australia	Postal districts, N = 267	i) Fast-food outlets (chain: Pizza Hut, McDonald's, Hungry Jacks, KFC, Red Rooster)	Median household income. Supplied in categories of weekly income: $160-199, $200-299, $300-399; $400-499, $600-699, $800-899. Collapsed into four categories due to only 5 postal districts in top two categories. SES 1: $400-899, SES2: $300-399, SES 3: $200-299, SES 4: $160-199.	i) Fast food outlet exposure increases as SES decreases.
Richardson, 2012 [78]	USA	Census block groups, N = 7588	i) Fast food outlets (chain and non-chain); ii) Grocery stores and supermarkets (chain and non-chain); iii) Convenience stores.	Neighbourhood poverty. Dichotomised into >20% or ≤20% of population below the federal poverty level. Neighbourhood minority. % of non-Hispanic white race/ethnicity categorised as low/medium/high but unclear how grouped. Created a categorical variable: low poverty/low minority, high poverty/low minority, low poverty/medium minority, high poverty/medium minority, low poverty/high minority, high poverty/high minority.	Findings were mixed. Descriptive data shows: i) In general, more fast food outlets in high-poverty compared to low-poverty areas, apart from in high-density urban medium-minority areas; ii) In general, more grocery stores/supermarkets in high-poverty compared to low-poverty areas, apart from in non-urban medium-minority areas and high-density high-minority areas; iii) Mixed findings for convenience stores depending on urban density and minority. For example, in both high-density urban areas, areas with a high minority population and high poverty have lower numbers of convenience stores on average, while in non-urban and low-density urban areas, those areas with a high minority population and high poverty have greater numbers of stores.
Rigby,2012 [79]	Leon County, Florida, USA	Census tracts, N = 48	i) Food stores; ii) Supplemental Nutrition Assistance Program (SNAP) accepting stores; iii) Supermarkets; iv) Grocery stores; v) Convenience stores; vi) Other stores (including supercentres, Dollar General stores, specialty food stores, pharmacies/drug stores, gasoline stations).	% of the population with income less than 100% of the federal poverty level. Dichotomised into low income (16.0-63.4%; n=24) and high income (0-15.2%; n=24).	i) Higher number of food stores in low income areas. No test for evidence of a difference; ii) A higher proportion of stores are SNAP accepting in low income compared to high income areas; iii) Proportion of SNAP accepting supermarkets greater in high income areas; iv) Proportion of SNAP accepting grocery stores was greater in low income neighbourhoods compared to high income neighbourhoods; v) Greater number of SNAP accepting convenience stores in low income neighbourhoods but no evidence of a difference; vi) A higher proportion of ‘other’ stores in low income areas were SNAP accepting than in high income areas. No evidence of a difference.
Schneider, 2013 [31]	Cologne, Germany	Social areas, N = 18	Relevant to food sales: i) Fast food outlets	Two measures of income: % of parents with joint income <€12,272; whether the district % of low-income parents was greater or less than 32% (the mean for the 269 social areas of Cologne).	i) Higher availability of fast food outlets as income decreases.
Sharkey, 2008 [80]	Texas, USA	Census block groups, N = 101	i) Food stores (supermarkets, grocery stores, convenience stores, discount stores, beverage stores, drug stores, specialty food stores); ii) Supermarket and grocery stores; iii) Convenience stores; iv) Discount stores.	Factor analysis of: neighbourhood unemployment; poverty; low education attainment; household crowding; public assistance; vehicle availability; telephone service. Split into three groups: low deprivation (lowest quartile), medium deprivation (middle 2 quartiles), and high deprivation (highest quartile of deprivation scores).	i) The distance to the nearest food store decreased with increasing deprivation. ii) - iv) Better access to supermarkets/grocery stores, convenience stores, and discount stores in more deprived areas.
Sharkey, 2009 [49]	Hidalgo County, USA	Census block groups, N = 197	i) Traditional food stores (supercentres, supermarkets, grocery stores); ii) Convenience food stores; iii) Non-traditional food stores (mass merchandisers: Kmart, Target, Wal-Mart, dollar stores, drug stores); iv) Fast food outlets.	Factor analysis of:neighbourhood unemployment;telephone service;public assistance;complete kitchen;complete plumbing;low educational attainment;poverty. One factor identified. The index was standardized by dividing by the square of the eigenvalue.	i) Distance to the nearest supermarket and grocery store increases with increasing deprivation. No evidence of an association between the number of supermarkets and grocery stores within one mile and deprivation. No evidence of an association between the number of supermarkets within three miles and deprivation. The number of grocery stores within three miles decreases with increasing deprivation. ii) No evidence of an association between distance to the nearest or the number of convenience stores within one mile or three miles and deprivation. iii) Distance to the nearest mass merchandiser, dollar store and pharmacy increases with increasing deprivation. No evidence of an association between the number of mass merchandisers, dollar stores, and pharmacies within one mile and deprivation but the number of each type within three miles decreases with increasing deprivation. iv) No evidence of an association between distance to the nearest fast food outlet and deprivation. Number of fast food outlets within one mile and within three miles decreases with increasing deprivation.
Sharkey, 2011 [81]	Central Texas Brazos Valley region, USA	Census block groups, N = 101	i) Fast food outlet; ii) Fast food opportunity (convenience stores); iii) Fast food opportunity with healthier entrees; iv) Fast food opportunity with a variety of healthier side dishes.	Based on: neighbourhood unemployment; poverty-level income; low educational attainment; household crowding; households receiving public assistance;households with no available vehicle; occupied housing with no telephone service. Split into three groups: low deprivation (lowest quartile of weighted and standardised deprivation scores), medium deprivation (middle two quartiles), and high deprivation (highest quartile).	i) High deprivation neighbourhoods had lower distance to nearest fast food outlet than low deprivation areas. The number of fast food outlets within three miles was higher in high deprivation neighbourhoods than in low deprivation areas. There was no evidence of an association between deprivation and the number of outlets within on mile. ii) High and medium deprivation neighbourhoods had lower distance to the nearest fast food opportunity than low deprivation areas. High deprivation neighbourhoods had more fast food opportunities within one and three miles than low deprivation areas. iii) High deprivation neighbourhoods had lower distance to the nearest fast food opportunity with healthier entrees than low deprivation areas. High deprivation areas had higher numbers of opportunities with healthier entrees within one and three miles than low deprivation areas. iv) High deprivation neighbourhoods had lower distance to the nearest fast food opportunity with healthier side dishes than low deprivation areas.High deprivation areas had higher numbers of opportunities with healthier side dishes within one and three miles than low deprivation areas.
Smith, 2010 [8]	9 sentinel sites in Scotland, UK	Data zones, N = 205	i) Food outlets; ii) Food outlets with at least one of twelve listed fruit and vegetables; iii) Large food outlet (>15,000 sq ft) with at least one of twelve listed fruit and vegetables; iv) Food retail outlet containing 1-4 fruit and vegetable items; v) Food outlet containing 5-8 fruit and vegetable items; vi) Food outlet containing 9-12 fruit and vegetable items.	Income sub-domain of Scottish index of multiple deprivation. Split into quintiles.	i) - iii) Travel times to food outlets, food outlets with fruit and vegetables, large food outlets with fruit and vegetables shorter in the most deprived compared to the least deprived areas. iv) & v) Median travel time to food outlet with 1-4 items and food outlet with 5-8 items shorter in the most deprived compared to the least deprived areas. vi) No evidence of an association between deprivation and travel time to food outlets containing 9-12 items.
Smoyer-Tomic, 2008 [56]	Edmonton, Alberta, Canada	Residential neighbour-hoods, N = 215	i) Supermarkets; ii) Fast food outlets.	Based on: low income; median income; unemployment; no high school diploma. Each grouped in tertiles.	i) Lower SES neighbourhoods were more likely to have a supermarket present within 800m than higher SES neighbourhoods. Only significant association identified when unemployment used to measure SES. ii) An area was more likely to have a fast food outlet present within 500m if it was of lower SES.
Svatisalee, 2011 [41]	Copenhagen, Denmark	Rodes, N = 388	i) Fast food outlets (chain and non-chain); ii) Supermarkets (chain and non-chain).	Low education; (used mean % as comparative cut-points) Average neighbourhood income (quartiles: low <€23,000, mid-low (€23,000-25,750), mid-high (€25,750-28,500), high (>€28,500).	i) Lower and middle income neighbourhoods had fewer fast food outlets than higher income neighbourhoods. ii) No evidence of an association between the number of supermarkets and neighbourhood SES.
Zenk, 2005 [7]	Detroit, USA	Census tracts, N = 869	i) Supermarkets (supercentres, national or regional chain).	% of residents below the poverty line. Split into tertiles (0-5.03%, 5.10%-17.2%, 17.23-81.96%).	i) Low income neighbourhoods had greater distance to the nearest supermarket than higher income neighbourhoods. Finding differ dependent on ethnicity.

*Measures are continuous predictors unless otherwise stated. *PCA: Principal Components Analysis. ^†^Numbers in the key findings column correspond to the number in the food store column.

### 3.2. Number of available food outlets

The most common type of outcome considered was the number of available food outlets within an administratively defined neighbourhood or a pre-specified buffer distance of the neighbourhood centroid, either geometric or population-weighted centroid [33]; 43 (79.6%) of the 54 articles considered this measure. These outcomes are counts as these can only be zero or positive whole numbers and, depending on the type of food outlet considered, potentially feature skewed distributions. For example, if the outcome is major fast food chain outlets within a small administrative unit the distribution is likely to be positively skewed, and potentially zero-inflated (have a larger number of zero values than assumed by a specific distribution), as many neighbourhoods will have only a small numbers of outlets while fewer neighbourhoods will have a large number. Thus, statistical approaches such as standard or zero-inflated Poisson and negative binomial regression which are equipped to deal with distributions of this nature are likely to be the most appropriate to use for this type of outcome.

The statistical methods adopted in the 43 articles which considered the number of outlets as an outcome are summarised in Table 2. Of these articles, only one third (n = 14) accounted for the fact that the outcome was a count through the use of Poisson [34]–[36] or negative binomial regression [9],[32],[37]–[41], Poisson multilevel regression [42] or generalised estimating equations [43], generalised additive models with Poisson errors [44], or a spatial scan statistical approach assuming a Poisson distribution [45]. Negative binomial regression is preferable to Poisson regression when the data are over-dispersed (i.e., when the variance is greater than the mean) as an assumption of the Poisson distribution is that the variance equals the mean. The negative binomial regression has an additional parameter which is able to deal with over-dispersed data and is often useful when the data are zero-inflated as can be the case in analyses of food outlet data. Of the analyses that assumed a Poisson distribution, two [36],[43] mentioned examining whether or not over-dispersion was present, finding no evidence of over-dispersion.

Other commonly used techniques which considered the outcome as a linear response variable included the one-way ANOVA or MANOVA (10 studies, 23.3%) or linear regression, whether single-level, multilevel or multivariate (6 studies, 14.0%). These techniques all assume that the residuals are normally distributed with mean zero and constant variance. In addition, these techniques assume that the observations are independent, apart from multilevel models which account for clustering in the data. Few studies mentioned considering the distributional assumptions in the analysis. Of the 16 studies, one log-transformed the outcome due to the skewed nature of the distribution [5] and one mentioned using the Kolmogorov-Smirnov test to determine if the assumption of normality was valid for their outcome variable, finding it to be reasonable [46]. Another article, while not discussing assessment of the outcome distribution, mentioned that the data were zero-inflated and thus presented a logistic regression analysis of the presence or absence of the outlet type in the neighbourhood [47]. However, a one-way ANOVA was used for the assessment of the association between the number of food outlets and neighbourhood-level SES. While ANOVA and linear regression can be robust to deviations from normality, the distribution of the number of food outlets would be more suitably dealt with using a method designed to deal with count data. Perhaps one reason for the use of these approaches is that the authors typically converted the food outlet outcome to a rate, either the number per 1,000 or 10,000 individuals, or the number per square mile or kilometre, thus converting a count outcome into a continuous variable prior to fitting the model. However, both Poisson and negative binomial regression are able to model rates by incorporating the log of population size or area as an offset in the model. Furthermore, rates are never negative and while techniques such as linear regression can yield expected values that are negative, those based on Poisson or negative binomial regression do not.

**Table 2.** Statistical methods used in articles which considered associations between the number of food outlets and neighbourhood SES (n = 43).

Method	Number of studies [ref. no.(s)]	Statistical software (n)	Adjusted for population and/or area	Assessed spatial auto-correlation
t-test	2 [31],[74]	Not reported (1), SPSS (1)	2	1
ANOVA	8 [6],[46],[47],[52],[69],[71]–[73]	SPSS (5), Minitab (1), Stata (1), Not reported (1)	7	0
MANOVA	2 [50],[68]	SPSS (2)	0	1
Kruskal-Wallis	2 [29],[79]	Stata (1), Not reported (1)	0	0
Correlation	6 [48],[66],[67],[70],[75],[76]	Not reported (5), SPSS (1)	2	1
Linear regression	3 [5]*[65],[78]	SPSS (2), Stata (1)	3	0
Multivariate regression	2 [49],[81]	Stata (2)	2	1
Ordered probit regression	1 [64]	Not reported (1)	1	0
Poisson regression	3 [34]–[36]	SAS (2), SPSS (1)	3	0
Negative binomial regression	6 [9],[37]–[41]	Stata (3), Not reported (1), R (1), SAS (1)	5	0
Generalised additive model with Poisson errors	1 [44]	S-Plus (1)	1	0
Negative binomial regression with clustered SEs	1 [32]	Stata (1)	0	0
Multilevel regression	1 [51]	HLM (1)	1	0
Multilevel Poisson regression	1 [42]	MLwiN (1)	1	0
Poisson regression with generalised estimating equations	1 [43]	SAS (1)	1	1
Bootstrap 95% confidence intervals and permutation test	1 [77]	Not reported (1)	1	0
Spatial scan statistic assuming Poisson distribution	1 [45]	SAS (1)	0	0
Not reported	1 [63]	Not reported (1)	0	0

* Log-transformed outcome; ** Includes Pearson and Spearman correlation

#### 3.2.1. Spatial autocorrelation

Only 5 (11.6%) of the 43 articles tested for evidence of spatial autocorrelation (Table 2); all used Moran's I. Values of spatial autocorrelation from Moran's I range from -1 to 1, with 0 indicating no correlation. Z-scores can be calculated for Moran's I values to determine whether or not there is evidence of spatial autocorrelation. However, evidence of spatial autocorrelation can also be determined using permutation tests which provide pseudo significance levels (i.e., pseudo *p*-values). These are classified as ‘pseudo’ since the significance is dependent on the number of permutations adopted. Permutation tests can be useful when assumptions underlying Moran's I tests, such as normality, are not appropriate.

Four articles examined spatial autocorrelation in the food outlet outcome variable(s) [31],[48]–[50] while one assessed residual spatial autocorrelation [43]. Of those that examined spatial autocorrelation in the outcome variable, one found no evidence of spatial autocorrelation but did not report estimates [31]. Another found no evidence of spatial autocorrelation (correlation = −0.04, pseudo-*p* = 0.46–0.49) in the number of grocery stores per acre, weak evidence of positive spatial autocorrelation of 0.11 (pseudo-*p* = 0.07–0.08) in the number of fast food outlets per acre, and evidence at the 5% significance level of positive spatial autocorrelation of 0.19 (pseudo-*p* = 0.01–0.02) in the number of convenience stores per acre [50]. In the third article, the authors reported evidence of spatial autocorrelation of 0.72 (z = 28.36) in the number of supermarkets within 1000m [48]. The fourth article reported positive spatial autocorrelation of between 0.25 and 0.62 (z = 5.66–13.53) for the number of supermarkets, 0.30 to 0.66 (z = 6.94-14.95) for the number of fast food restaurants and 0.29 to 0.41 (z = 6.32–9.73) for the number of convenience stores depending on the buffer size used to define the neighbourhood, with correlation increasing as the buffer increased from 1 to 5 miles [49]. Two of the articles which found evidence of spatial autocorrelation in the outcome did not account for this in the analysis or test for evidence of residual spatial autocorrelation after examining the associations with neighbourhood-level SES [48],[49]. Thus, the results from the analyses may have been affected if residual spatial autocorrelation remained. The third article which found evidence of spatial autocorrelation in the outcome conducted bivariate spatial autocorrelation analyses of the food outlet outcome alongside neighbourhood-level SES in order to determine associations [50]. However, the analytical results presented were based on the use of MANOVA which does not take into account the spatial location of the neighbourhoods. Lisabeth et al. (2010) examined residual spatial autocorrelation after fitting a multivariate Poisson regression using generalised estimating equations to deal with the clustering of the different stores within census tracts and found no evidence of residual spatial autocorrelation (all *p*-values > 0.37). However, the authors stated that evidence of residual spatial autocorrelation was identified when using buffer sizes to define neighbourhoods rather than census tracts and thus the estimates of the standard errors from that analysis were not valid. No attempt to incorporate the spatial information about the data was made to account for this residual spatial autocorrelation.

#### 3.2.2. Spatial methods

Although only 5 articles explicitly tested for spatial autocorrelation in the analysis, others incorporated information about the spatial location of the data in different ways. For example, three articles considered the clustering of small administratively defined neighbourhoods within larger area level definitions, such as local authorities or counties, using multilevel modelling [42],[51] or clustered standard errors [32]. While these methods deal with the grouping of neighbourhoods, they do not explicitly examine the spatial location as such, in that neighbourhoods could have similar observations to those they surround and these neighbourhoods will not be located within the same local authority or county if they are at the edge of these administrative levels. One analysis included the spatial location as a covariate in the analysis in order to potentially account for any spatial autocorrelation [44]. Residual spatial autocorrelation was not examined in any of these articles.

Only one of the 43 articles adopted a spatial analytical technique to examine associations between the number of food outlets and neighbourhood-level SES. In this analysis, Baker et al. adopted a spatial scan approach in which a circular window of a pre-defined radius is moved across the map to test the null hypothesis that the rate of food outlets is the same in all of the windows, assuming a Poisson distribution for the outcome variable [45]. This technique identifies clusters in which higher or lower rates are observed than expected and adjustment for neighbourhood-level SES can be examined to determine if this explains these clusters.

A small number of studies mention the lack of consideration of spatial autocorrelation in the study limitations [36],[44],[52].

### 3.3. Distance to the nearest food outlet

Fourteen (25.9%) of the 54 articles considered distance to the nearest food outlet as the accessibility measure, shown in Table 3. Although Hurvitz et al. considered this outcome in addition to the density of outlets, no formal statistical analysis was conducted of the association between the distance and neighbourhood-level SES [38]. Of the fourteen articles, eleven (78.6%) feature in Table 2 as these studies also considered the number of outlets as an outcome measure. Ten of these used the same statistical methods for both the count measure and the distance measure. The most common techniques used were the one-way ANOVA (4 articles, 28.6%) or linear regression, including multivariate linear regression, (4 articles, 28.6%). Although these techniques are perhaps more appropriate for distance measures, it is possible that these types of measures could be skewed. Most articles did not mention any assessment of the shape of the distribution or examination of model residuals. In one article, the distance outcome was log-transformed to obtain a normally distributed outcome variable [40]. Another analysis, although using ANOVA, reported median distances suggesting that the data were skewed [53].

**Table 3.** Statistical methods used in articles which considered associations between distance to the nearest food outlet and neighbourhood SES (n = 14).

Method	Number of studies [ref. no.(s)]	Statistical software (n)	Adjusted for population and/or area	Assessed spatial auto-correlation
t-test	1 [31]	SPSS (1)	0	1
ANOVA	4 [52],[53],[72],[73]	SPSS (2), Stata (1), Not reported (1)	4	0
Kruskal- Wallis	1 [29]	Not reported (1)	0	0
Correlation	3 [48],[67],[70]	Not reported (3)	2	1
Linear regression	2 [40],[80]	Stata (2)	2	0
Multivariate regression	2 [49],[81]	Stata (2)	2	1
Moving average spatial regression	1 [7]	S+SpatialStats (1)	1	1

#### 3.3.1. Spatial autocorrelation

Four (28.6%) of the 14 articles examined spatial autocorrelation using Moran's I [7],[31],[48],[49] (Table 3), three of which also assessed spatial autocorrelation for the count of food outlets (Table 2). Three articles only considered spatial autocorrelation in the outcome [31],[48],[49], while one examined residual spatial autocorrelation [7]. Considering spatial autocorrelation in the food outlet distance outcome, one article found evidence of positive spatial autocorrelation of 0.54 (z-score = 21.68) in the distance to the nearest supermarket [48]. Another found varying degrees of positive spatial autocorrelation dependent on the outlet type, ranging from 0.20 (z-score = 4.51) for distance to the nearest fast food restaurant to 0.70 (z-score = 15.17) for distance to the nearest mass merchandiser. The spatial autocorrelations for the distance to the nearest supermarket and nearest grocery store were 0.50 (z-score = 10.41) and 0.61 (z-score = 13.57), respectively [49].

Although these analyses found evidence of spatial autocorrelation in the outcome, neither tested for residual spatial autocorrelation when modelling associations with neighbourhood-level SES, nor took the spatial location into account in the analysis. Schneider and Gruber mentioned that they found no evidence of spatial autocorrelation in the outcome, although they did not explicitly mention testing this for the distance accessibility measure, only the count measure [31]. Zenk et al. found evidence of residual spatial autocorrelation (Moran's I = 0.008, *p* < 0.001) after fitting ordinary least squares regression and thus used a moving average spatial regression analysis to account for any spatial autocorrelation present in the residuals [7].

Moving average spatial regression, unlike ordinary least squares regression, allows for spatial autocorrelation in the residual terms by taking the spatial location of the neighbourhoods into account. This form of spatial regression considers the influence of local neighbours; that is, it is assumed that observations in one neighbourhood are directly influenced by observations in the closest neighbourhoods but not in the neighbourhoods beyond. In order to fit a moving average spatial regression, it is necessary to define a neighbours matrix to describe the spatial relationships in the data. If, for example, the study region involves 100 administrative units, the neighbours matrix will be a square matrix with 100 rows and 100 columns to represent all of these units. The diagonal entry of the matrix will equal zero as administrative units cannot neighbour themselves. If two administrative units are neighbours then an entry of 1 will be included in the matrix; an entry of 0 indicates that the two units are not neighbours. Commonly, two administrative units are defined as neighbours if they share a common boundary. Although, alternatively, neighbours could be defined according to distance measures (e.g., defining areas to be neighbours if the distance between the administrative unit centroids is less than 2km). Zenk et al. did not describe how the neighbours matrix was created but mentioned that accounting for the spatial structure of the data using moving average spatial regression resulted in no remaining residual spatial autocorrelation.

#### 3.3.2. Spatial methods

None of the other eight articles considered spatial autocorrelation or spatial analytical methods, although, as with the count outcome, one did examine whether including clustered standard errors affected the results, reporting them to be similar to the results without clustered standard errors [52].

### 3.4. Alternative food outlet accessibility measures

Of the eight articles not discussed in sections 3.2 and 3.3, three considered travel time in minutes to the nearest food outlet [8],[54],[55]. In each study, the authors acknowledged that the travel times were skewed and, thus, not normally distributed. One analysis adopted linear regression [55] and two used Spearman's rank order correlation [8],[54]. None of these articles mentioned which statistical software package they used in the analysis or discussed spatial autocorrelation.

One article considered two binary outcomes- the presence or absence of fast food outlets within 500m or supermarkets within 800m from the geometric centroid of each census block- and fitted logit models in Stata to examine associations with neighbourhood-level SES [56]. Another considered travel times from each census block to the nearest supermarket or fast food outlet and then categorised each census block as either having a shorter time to a supermarket, a shorter time to the fast food outlet, or the same time to each outlet [57]. Using categories rather than actual distance values led to a loss of information about the magnitude of the differences in distance, making it difficult to determine how access to these outlet types could differ. To examine associations between neighbourhood-level SES and these categories of access, the authors fitted a one-way ANOVA of continuous SES score (Socioeconomic Index for Areas, SEIFA).

In a third article, a composite measure of food outlet access was derived by assigning neighbourhoods with a score of 1 for each of three different healthy and three different unhealthy outlets if located within a quarter mile network area [58]. Thus, each neighbourhood would have a score between 0 and 3 for healthy outlets and for unhealthy outlets. This measure is limited in that the scores do not take into account the number of outlets within a neighbourhood (e.g., a score of one is assigned to the neighbourhood regardless of whether it has one supermarket or ten within a quarter mile). The healthy outlet score was subtracted from the unhealthy outlet score to give a range of scores from -3 to 3 for the neighbourhoods considered. This outcome is difficult to interpret given that, for example, a score of zero for neighbourhoods which have neither healthy nor unhealthy outlets within a quarter mile cannot be distinguished from a score of zero for neighbourhoods which have three healthy and three unhealthy outlet types. The association between the outlet score and neighbourhood-level SES was assessed using one-way ANOVA in SAS, although the authors did not mention assessing the shape of the outcome distribution. None of these three studies assessed or mentioned the possibility of spatial autocorrelation in their data.

The other two studies adopted spatial analytical approaches in the analysis [30],[59]. Dai and Wang used a spatial lag model to examine the distribution of weight scaled food outlet accessibility measures (using weights of 0–10 based on name recognition) by neighbourhood-level SES variables [59]. A spatial lag model incorporates a weighted average of the outcome values of neighbouring observations into a regression model to remove any residual spatial autocorrelation. In a spatial lag model, a neighbours matrix (as described previously) is required. The spatial lag term is created by multiplying the neighbourhood matrix (typically standardised so that the sum of each row is equal to one) by the outcome variable (i.e., the food outlet outcome). Typically, many of the terms in the neighbourhood matrix are 0 as a lot of neighbourhoods do not neighbour one another. Therefore, for each neighbourhood, the spatial lag term is the weighted average of the observations in the immediately surrounding neighbourhoods. An alternative to the spatial lag model is the spatial error model (although it is possible to incorporate both a spatial lag and a spatial error term in a model) which takes into account the location of observations by modelling the correlation in the error term. In the absence of any clear view as to which is the more appropriate structure to model, model comparison techniques can be adopted to aid in deciding which captures the underlying spatial structure of the data [60],[61].

Lee and Lim adopted a more complex food outlet accessibility measure by deriving a discrepancy index, in which they calculated the expected demand for an outlet and divided this by the observed number of outlets in the neighbourhood [30]. A ratio of 1 indicates that there are sufficient outlets in the neighbourhood, while < 1 indicates that there is an over-supply in the neighbourhood, and >1 indicates that the demand is greater than the supply. The authors used the G-statistic to examine the spatial distribution of the outcome. The G-statistic aids in the identification of clusters and tests the null hypothesis that there is no clustering of the variable of interest, the discrepancy index in this case; that is, there is complete spatial randomness in the distribution of the variable. The G-statistic estimates the spatial clustering of values of environmental features. The statistic takes high values where higher values of the observations cluster and low values where lower values of the observations cluster [62].

### 3.5. Summary

Only five articles (9.3%) included in this review adopted a spatial statistical technique in the analysis of the equity of access to food outlets, each using a different technique. These methods were: moving average spatial regression [7], spatial scan statistic [45], G-statistic [30], spatial lag model [59], and bivariate spatial autocorrelation assessment [50]. A sixth study incorporated the spatial location of neighbourhoods in a regression model [44]. Seven (13.0%) of the 54 studies tested for spatial autocorrelation, while only a further three mentioned spatial autocorrelation at all.

## 4. Discussion

A number of systematic reviews have considered the evidence supporting inequities in access to food outlets. While these reviews discussed differences between studies in terms of the definitions of access, neighbourhood SES and the neighbourhood boundaries or buffers adopted, none explicitly examined the statistical methodologies employed.

Our review has shown that a variety of methods have been employed to examine the equity of food outlets by deprivation, with 17 analytical techniques used to determine associations between the number of food outlets and neighbourhood-level SES and seven techniques used to test for associations between the distance to the nearest outlet and neighbourhood-level SES. It is not possible for us to determine how findings may have been affected by the analytical approach as this will be dependent on a number of factors including, for example, the sample size of the study and the validity of the model assumptions. While the assumption of normality, and thus the use of linear regression, t-tests or ANOVAs, may be valid for large sample sizes, it is important to consider precisely what question is being asked and whether the approach utilised is appropriate [82]. In this area of research, commonly the number of food outlets was considered as an outcome variable. This is a count variable, only taking zero and positive integer values. Therefore, the normal distribution, which assumes an equal distribution around the expected value (either positive or negative), is not the most appropriate for dealing with data of this type. Count variables are more suited to analyses using Poisson or negative binomial regression. Although we focussed particularly on the treatment of the outcome variable in these analyses, it is worth noting that treatment of the exposure variable should not be overlooked. In particular, there is often a tendency to adopt arbitrary percentile categorisation of exposure variables (discussed elsewhere [83]).

When considering analyses of the availability of food outlets by small-area level deprivation, it is important to acknowledge that these studies involve spatial data and thus this feature should also be considered when determining the statistical approaches to employ in the analysis. Our systematic review has shown that this feature is infrequently considered in studies of the equity of outlets with most relying on traditional regression techniques which assume that the residuals are independently distributed; an assumption which should be verified when dealing with spatial data. Thus, it was unclear whether residual spatial autocorrelation remained which could affect the inference from the models. Furthermore, studies which found evidence of spatial autocorrelation infrequently adopted spatial regression techniques to attempt to model the spatial autocorrelation. It therefore appears that there may some confusion within this field of research about how spatial data can and should be dealt with in analyses. It is possible to draw on examples looking at the distribution of other facilities which have considered the spatial nature of the data [84],[85].

One potential reason for the lack of consideration of the spatial nature of the data, other than a possible unfamiliarity with the problems associated with ignoring spatial autocorrelation, may be due to the functionality of software used to map data or the users' familiarisation with the capabilities of this software. Typically GIS software packages such as ArcGIS were adopted to map the data and determine the number within a given region, before transferring the data to a statistical software package to determine if neighbourhood-level SES was associated with the food outlet outcome measure. In transferring the data to the statistical software package, it is likely that the spatial aspects of the data were not retained for consideration in the analysis. Dealing with spatial data can be non-trivial. However, commonly used software packages, such as SAS [86], Stata [87],[88] and R [89], offer options for conducting spatial analysis. Other spatial analytical software is available which could be used in studies of this nature. Notably, studies in this review which tested for spatial autocorrelation either used ArcGIS/ArcView or a specialist spatial analytical package for this purpose, such as GeoDa [90] or S + SpatialStats [91]. However, in those studies that employed GeoDa, other statistical software packages, such as SPSS or Stata, were used to test for associations between neighbourhood-level SES and food outlet outcome even though GeoDa does provide some options for regression models.

Another possible reason for not considering spatial autocorrelation or spatial regression techniques in these analyses may be due to the number of neighbourhoods considered in some studies. The larger studies discussed in this review consisted of several thousand observations meaning that large neighbours matrices are required in order to determine the level of spatial autocorrelation or to fit spatial regression models. This can prove to be computationally intensive. However, various techniques have been proposed to deal with large spatial data sets, including techniques involving sparse matrix operations, in which only the non-zero elements of the neighbours matrix are stored [92],[93]. Some studies may not have considered spatial autocorrelation or spatial techniques as the areas considered were not spatially contiguous. However, spatial neighbours do not necessarily have to be defined as those which share a common boundary; distance based definitions of neighbours can be used but this poses the question as to what distance should be used.

There are a number of analytical techniques which can deal with spatial data. These include spatial regression techniques which are able to model associations between areal measures, such as the number of food outlets within a neighbourhood and neighbourhood-level SES, while accounting for the spatial nature of the data. One such approach is the spatial moving average regression described previously, adopted by Zenk et al. [7], which enables the spatial autocorrelation in the data to decline rapidly beyond direct neighbours [94]. In spatial epidemiology or ecology literature dealing with areal data, often conditional autoregressive (CAR) [95]–[97] or simultaneous autoregressive (SAR) [98]–[100] models are used. Spatial auto-regression models expand on traditional regression models through the creation of a spatial dependence between the outcome observations (e.g., the number of food outlets) or the residuals at neighbouring locations through the use of a weighted neighbours matrix (described previously). This matrix specifies the strength of the interaction between the neighbouring units [28],[101],[102]. Choosing an appropriate spatial model to adopt in the presence of spatial autocorrelation can be challenging and requires some care [103].

Other approaches, such as the spatial scan statistic or the G-statistic, are useful for detecting clusters of higher or lower availability of food outlets. Alternative clustering techniques have been proposed in other food environment literature, such as the bivariate K-function [104]. However, this approach has received criticism as to its appropriateness in built environment studies [105].

Clearly, the technique to employ is dependent on the research question being posed and the underlying nature of the spatial data. Dealing with spatial data is by no means trivial. Therefore, care should be taken to ensure the validity of the assumptions imposed by the modelling adopted.

### Limitations of the review

Our search strategy was limited to articles published in the English language and, thus, may not have included all relevant papers. While it is beyond the scope of this review to discuss in depth the numerous spatial analytical approaches available, we hope that highlighting possible approaches to account for the spatial nature of the data aids future analyses in this field.

## 5. Conclusion

While researchers continue to explore the impact of the neighbourhood environment on disadvantaged groups in society through the examination of the equity of access to food outlets, it is important to highlight that results may differ dependent on the analytical approach adopted, particularly given the spatial nature of the data. While much detail is usually provided on the data collection and mapping using GIS software, the description of statistical procedures is often brief and lacks sufficient information. It is recommended that future studies consider the validity of the assumptions underlying the analytical approach adopted and assess the residual spatial autocorrelation following standard modelling, adopting spatial analysis techniques where appropriate.

## Appendix

### S1: Search strategy and results

Database provider and databases

The following databases were chosen to provide a comprehensive search of relevant peer-reviewed literature:

EBSCOhost: Medline Complete
EBSCOhost: PsychINFO
EBSCOhost: CINAHL Complete
Web of Science: Social Sciences
Global Health: Ecology & Environmental Sciences; Agricultural Economics & Rural Studies; Human Sciences; Leisure, Recreation & Tourism
Embase: Epidemiology; Prevention
Scopus: Health Sciences; Social Sciences & Humanities
Cochrane Library

Search terms

We identified key search terms using existing systematic reviews of access to food outlets and literature identified from a smaller scoping search conducted previously which used the following search terms generated by authors: “food outlets”, “food stores”, “amenities”, “fast food”, “supermarkets” (terms related to the outcome); “deprivation”, “SES” (terms related to the predictor); “neighbourhood”, “neighborhood”, “area” (terms related to the level of the analysis). This led to an extensive list of possible search terms shown in the table below.

**Table A1.** Possible search terms for review of equity of access to food outlets.

	Possible terms
**Outcome**	
Food outlets	“Food”, “Fast food”, “Fruits”, “Vegetables”, “Supermarket”, “Food environment”, “Food desert”, “Food access”, “Food accessibility”, “Food supply”, “Food stores”, “Food outlets”, “Fast food outlet”, “Fast-food outlet”, “Food retailing”, “Fruit and vegetable supply”, “Retail outlets”, “Community resources”
**Predictor**	
Area-level deprivation	“Socioeconomic”, “Inequality”, “Inequalities”, “Socio-economic disadvantage”, “Area-level disadvantage”, “Deprivation”, “Material deprivation”, “Area deprivation”, “Socio-economic status”, “Socioeconomic status”, “Socioeconomic factors”, “Disparities”, “Health disparities”, “Health status disparities”, “Socio-economic inequality”, “Poverty areas”, “Social class”, “Social determinant”
**Spatial scale**	
Neighbourhood	“Neighbourhood”, “Neighborhood”, “Environment”, “Residence”
**Access**	
	“Spatial accessibility”, “Access”, “Accessibility”, “Availability”
**Other relevant terms**	
	“Geographic Information System”, “Geographical Information System”, “GIS”, “Mapping”, “Geographic mapping”, “Spatial analysis”, “Spatial patterning”, “Spatial clustering”, “Spatial autocorrelation”

Using this information, the authors compiled a final list of key search terms to be entered into the search field of each database. These were separated into three categories: a) food outlets, b) equitable, c) neighbourhood access and database searches of a) AND b) AND c) were conducted. The complete list of search terms were:

- **Food outlets**
  “fast foo*”, fast-foo*, frui*, vegetabl*, supermarke*, “food environmen*”, “food deser*”, “food suppl*”, “food stor*”, “food outle*”, “food retai*”, “fruit and vegetable suppl*”, “groce*”, “greengrocer*”, “green groce*”, green-groce*, “convenience stor*”, takeaway, “take-away”
- **Equitable**
  equit*, inequit*, socioeconomic*, socio-economi*, equalit*, inequalit*, advantag*, disadvantag*, deprivation, disparit*, “social class”, “social determinan*”
- **Neighbourhood access**
  neighbourhoo*, neighborhoo*, environment*, acces*, availab*, distribution, location

Refining searches

Searches in the above databases were limited (where the database allowed) to peer-reviewed English language articles published since 2000. Truncated terms were used where appropriate such as for “equity or equities”, “neighbourhood or neighbourhoods”. Wildcard terms were used where appropriate for words that had different spelling (e.g., to search for neighbourhood or neighborhood, the term “neighb$rhood” was used).

Search results

The specific search terms, strategies and approaches used in each database, and the numbers of resulting articles identified, are provided below.

**Table A2.** Search results by database.

Database Provider	Database	Limiters	Search string	Results	Search date
EBSCOhost	Medline Complete	2000–2014 English Academic Journals	a) **AND** b) **AND** c)	2117	12/3/14
EBSCOhost	PsychINFO	2000–2014 English Academic Journals	a) **AND** b) **AND** c)	350	12/3/14
EBSCOhost	CINAHL Complete	2000–2014 English Academic Journals	a) **AND** b) **AND** c)	544	12/3/14
Web of Science	Social Sciences	Topic search 2000–2014 English Academic Journals	a) **AND** b) **AND** c)	1298	12/3/14
Global Health	Ecology & Environmental Sciences	2000–2014 English Academic Journals	a) **AND** b) **AND** c)	1295	13/3/14
	Agricultural Economics & Rural Studies				
	Human Sciences				
	Leisure, Recreation & Tourism				
Embase*	Epidemiology Prevention	2000–2014 English Academic Journals	a) **AND** b) **AND** c)	697	13/3/14
Scopus	Health Sciences Social Sciences and Humanities	2000–2014 English Academic Journals	a) **AND** b) **AND** c)	1346	13/3/14
Cochrane Library		2000–2014	a) **AND** b) **AND** c)	2	13/3/14

*(‘fast food’ OR ‘fast foods’ OR ‘fast-food’ OR ‘fast-foods’ OR fruit OR fruits OR vegetable OR vegetables OR supermarket OR supermarkets OR ‘food environment’ OR ‘food environments’ OR ‘food desert’ OR ‘food deserts’ OR ‘food supply’ OR ‘food supplies’ OR ‘food store’ OR ‘food stores’ OR ‘food outlet’ OR ‘food outlets’ OR ‘food retail’ OR ‘food retailing’ OR ‘fruit and vegetable supply’ OR ‘fruit and vegetable supplies’ OR grocer OR grocery OR grocers OR greengrocer OR greengrocers OR ‘green grocer’ OR ‘green grocers’ OR green-grocer OR green-grocers OR ‘convenience store’ OR ‘convenience stores’ OR takeaway OR take-away)

AND (equity OR inequity OR equities OR inequities OR socioeconomic OR socio-economic OR equality OR equalities OR inequality OR inequalities OR advantage OR advantages OR disadvantage OR disadvantages OR deprivation OR disparity OR disparities OR ‘social class’ OR ‘social determinant’ OR ‘social determinants’)

AND (neighbourhood OR neighbourhoods OR neighborhood OR neigborhoods OR environment OR environments OR access OR accessibility OR available OR availability OR distribution OR location)

### S2: Data extraction

General data extraction

Study details (title, authors, country); study characteristics (aims; area-level measure and number of areas; study type, i.e. cross-sectional or longitudinal); outcome measure (type of food store; data source; count, distance, density); predictors (measure of area-level deprivation; ethnicity; other covariates); statistical analysis (methods used; spatial autocorrelation assessed; software); results; conclusions drawn by authors.
